# Improving Refugee Children: A Randomized Controlled Trial on the Impact of Cognitive Activity Training

**DOI:** 10.1186/s40359-025-03586-z

**Published:** 2025-10-28

**Authors:** Sümeyye Belhan Çelik, Gonca Bumi̇n

**Affiliations:** 1 Occupational Therapy, Faculty of Hamidiye Health Sciences, University of Health Sciences, Istanbul, Türkiye; 2 Occupational Therapy, Faculty of Health Sciences, Hacettepe University, Ankara, Türkiye

**Keywords:** Refugees, Cognitive function, Cognitive training, Academic performance, Occupational therapy

## Abstract

**Backround:**

Refugee children’s cognitive skills are negatively impacted by war and displacement, reducing school performance. Addressing these challenges requires developmentally appropriate and function-oriented approaches that align with occupational therapy’s focus on participation and skill-building. It was aimed to examine the effects of Cognitive Activity Training (CAT) on cognitive skills, academic performance and quality of life in refugees.

**Methods:**

The study was designed as a randomized controlled study, including pre and post testing. A total of 34 refugee children in study group (14.29 ± 0.84years) and control group (14.41 ± 0.61years) were included. Children in the study group received the CAT program (10 weeks, 2 sessions per week), based on occupational therapy principles and implemented as part of a cognitive rehabilitation approach. Children in the control group received no intervention. Refugee children were assessed before and after the intervention using the Loewenstein Occupational Therapy Cognitive Assessment (LOTCA), Reading Speed Test (RST), Minnesota Handwriting Test (MHT), and Pediatric Quality of Life Inventory (PedsQL).

**Results:**

It was found that the school-based CAT given to the refugee children had a statistically significant effect on all cognitive skill parameters, reading speed and handwriting skills reflecting academic performance, and quality of life (*p* < .05). ANCOVA results showed that post-test adjusted means of LOTCA Total and LOTCA Visual Perception scores were significantly higher in study group compared to control group (*p* < .001). Similarly, Mixed Design ANOVA indicated significant interaction effects in cognitive skill subdomains, academic performance measures (RST and MHT), and PedsQL total and subdomain scores (*p* < .001). While there were no significant differences between pre-test scores of study and control groups, study group demonstrated significant within-group improvements post-intervention, whereas control group showed no significant changes.

**Conclusion:**

This study demonstrated that CAT may support cognitive, academic, and quality of life outcomes in refugee children exposed to war. Thus, cognitive skills should be assessed in schools for refugee children and cognitive activity training to be applied within the scope of rehabilitation should be included in occupational therapy programs for this population.

**Trial registration:**

ClinicalTrials.gov identifier: NCT05093738- 09/ 28/ 2021 (https://clinicaltrials.gov/study/NCT05093738?id=NCT05093738&rank=1).

**Supplementary Information:**

The online version contains supplementary material available at 10.1186/s40359-025-03586-z.

This article is extracted from Sümeyye Belhan Çelik's doctorate dissertation entitled "Investigation of the Effects of School-Based Cognitive Activity Training on Cognitive Skills, Academic Performance and Quality of Life in Refugee Children", supervised by Gonca Bumin (Ph.D. Dissertation, Hacettepe University, Ankara, 2022).

## Introduction

The term “refugee” refers to individuals who, due to fear of persecution based on ethnicity, race, religion, or political opinion, leave their country of origin and seek protection elsewhere [1]. Migration, regardless of legal status, brings significant disruptions to the lives of individuals and future generations. The Syrian civil war, which began in 2011, has led to one of the largest forced migrations in recent history, affecting both sending and receiving countries [2]. According to the Presidency of Migration Management, as of 2024, Türkiye hosts over 3.4 million Syrian refugees, the highest number worldwide [3, 4].

Refugee children are among the most vulnerable groups affected by conflict and forced migration. Traumatic and adverse environmental conditions experienced before, during, and after forced migration—including war exposure, violence, and displacement—contribute to the development of mental health problems in displaced populations [5]. By the end of 2023, the population of Syrian immigrant children aged 0–18 in Türkiye reached 1,638,310, indicating that about one-third of immigrants in Türkiye are of school age [4]. In addition to high rates of anxiety, depression, and post-traumatic stress disorder (PTSD), refugee children face significant challenges in academic achievement and cognitive functioning [6–8].

PTSD, the most frequently observed trauma-related condition among refugee children, is associated with symptoms such as attention deficits, memory problems, and emotional dysregulation, all of which hinder school participation and learning [9, 10]. These symptoms can lead to disruptions in executive functioning, working memory, and sustained attention, making it difficult for refugee children to meet the cognitive demands of the classroom [14].

Several studies have documented the association between trauma exposure and academic underperformance in refugee children. For instance, research on Palestinian refugee children has shown that the severity of war- and migration-related trauma is negatively correlated with academic success and quality of life [11]. However, despite this evidence, there is a notable lack of intervention studies specifically targeting the cognitive development of refugee children [12].

Structured cognitive rehabilitation programs offer a promising avenue for addressing these challenges. These programs aim not only to restore impaired cognitive skills but also to strengthen existing abilities through systematic, repetitive practice. Cognitive rehabilitation is generally described in terms of two complementary approaches. The remedial (or restorative) approach focuses on directly training specific cognitive domains such as attention and memory through structured, table-based exercises, digital applications, and repetition-based tasks. In contrast, the functional approach emphasizes enhancing everyday functioning by using compensatory strategies and making environmental adaptations to support cognitive performance in real-life settings [13].

According to existing literature, cognitive training is considered a promising approach to support cognitive development and academic functioning in children and may offer benefits for those affected by adversity, such as refugee populations [14–16]. It is also considered a priority to plan interventions for the cognitive skill areas where weaknesses are observed, and to gain a clearer understanding of refugee children’s capabilities and the barriers they face in school and academic performance.

However, no intervention study involving cognitive training had been found when the literature was examined, and cognitive analyses and evaluations were conducted on the refugee population [14]. The hypotheses of our study, which aimed to investigate the effects of school-based cognitive activity training on cognitive skills, academic performance, and quality of life in refugee children, are as follows:

- H₀: Cognitive activity training has no significant effect on refugee children’s cognitive skills, including the domains of attention, memory, visual perception, visuomotor organization, praxis, spatial perception, and thinking operations.
- H₁: Cognitive activity training has a significant effect on refugee children’s cognitive skills, including the domains of attention, memory, visual perception, visuomotor organization, praxis, spatial perception, and thinking operations.
- H₀: Cognitive activity training has no significant effect on refugee children’s academic performance, including the domains of reading speed and writing skills.
- H₁: Cognitive activity training has a significant effect on refugee children’s academic performance, including the domains of reading speed and writing skills.
- H₀: Cognitive activity training has no significant effect on refugee children’s quality of life, including emotional, social, school, physical health, and psychosocial domains.
- H₁: Cognitive activity training has a significant effect on refugee children’s quality of life, including emotional, social, school, physical health, and psychosocial domains.

### Study sample and participant characteristics

Thirtyfour children, seventeen in each group (study and control) were included in the study. The study inclusion criteria were being between the ages of 13 and 16, being literate in Turkish, having war experience in their country, and elevated post-traumatic stress symptoms (score of 12 or more on the Child Post Traumatic Stress Disorder Reaction Index Scale, CPTS-RI), scoring below threshold on a cognitive test (less than 21 points in the Montreal Cognitive Assessment Scale, MoCA) and consenting to participate in the study. The study did not include children who had any neurological, psychiatric, orthopedic diagnoses.

Between 16 March 2021 and 12 May 2021, a total of 82 children were interviewed for this study at a single public secondary school located in Istanbul, Türkiye, and 48 children were excluded because they did not meet the inclusion criteria [17]. CPTS-RI was used a 20-item Likert-type scale assessing post-traumatic stress symptoms in children. The Turkish adaptation was conducted by Erden et al. Scores of 12 and above were used to determine eligibility for inclusion. Another inclusion criterion was the MoCA. The reliability and validity of the Turkish adaptation of the MoCA was conducted in 2014 [18] It was used to identify children with cognitive impairment, with scores below 21 considered indicative of reduced cognitive functioning. They were administered only at the initial stage of participant screening.

The most common reasons for ineligibility were as follows: 22 children scored below 12 on the CPTS-RI, indicating no significant post-traumatic stress symptoms; 18 children scored 21 or higher on the MoCA, suggesting relatively preserved cognitive functioning; and 8 children were excluded for being outside the targeted age range, being illiterate in Turkish, or having no history of direct war exposure. These inclusion criteria ensured that the final sample consisted specifically of refugee children with both cognitive vulnerability and significant trauma exposure.

**Fig. 1:**
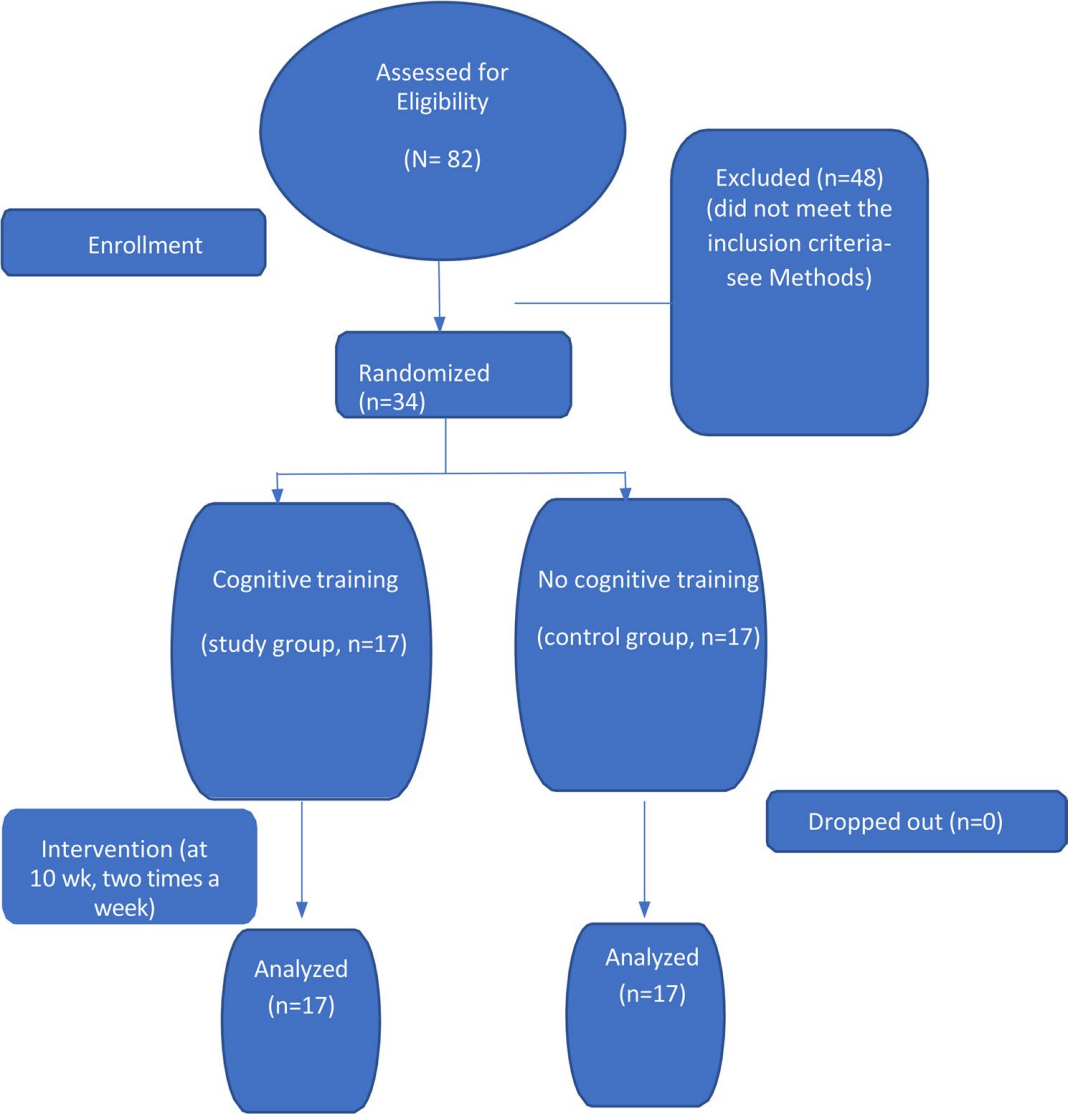
CONSORT flow diagram: the flow of children during each trial phase

Based on the sample size determined using G*Power analysis, participants were randomly assigned by using the simple randomization method to the study group (*n* = 17) and control group (*n* = 17) [19] (Fig. 1). The random allocation sequence was generated by an independent researcher, while participant enrollment and assignment to interventions were carried out by the principal investigator.

After the scope and purpose of the study were explained in detail, informed consent was obtained from the participants; as they were under the age of 16, written consent was also obtained from their parents or legal guardians. The study protocol was approved by the Hamidiye Non-invasive Investigation Ethics Committee (25 April 2020/20–33). Furthermore, this study was registered by the Registry of Clinical Trials (NCT05093738).

### Experimental design and assessment procedures

The demographic and clinical characteristics of the participants regarding their age, gender, class they are in, how many years ago they came to our Türkiye were recorded using the demographic information form prepared by the researchers. Loewenstein Occupational Therapy Cognitive Assessment (LOTCA), Reading Speed ​​Test (RST), Minnesota Handwriting Test (MHT), and the Pediatric Quality of Life Inventory (PedsQL) were administered pre- and post-intervention to evaluate changes across cognitive, academic, and quality of life domains.

LOTCA Version 2, a tool that practically evaluates cognitive skills was developed by Itzkovich in 2000 [20]. There are 26 items in total in the evaluation method, which consists of 6 sub-dimensions. The sub-dimensions are orientation (2 items), visual perception (4 items), spatial perception (3 items), praxis (3 items), visual-motor organization (7 items) and thinking processes (7 items). Test materials mainly consist of decks of cards, colored blocks, pegboard set and other materials. According to the application instruction, the scores obtained from each test parameter range from 1 to 4, and high scores indicate higher cognitive functionality. The total score ranges from 26 to 115 [21].

The reading and writing parameters were examined with *RST* and *MHT* to evaluate the academic performance of children. RST was used to evaluate reading. In our study, “academic performance” was operationalized through fundamental literacy components, namely reading fluency and handwriting legibility, given the feasibility constraints and the targeted age group. In this context, a standard text consisting of 313 words was selected from school textbooks appropriate to the children’s level. Each child was asked to read the text aloud individually during both the pre-test and post-test sessions. Reading speed was calculated using the following formula: (total number of words/reading time in seconds) × 60 (237). The MHT, developed by Reisman is one of the standardized tests with proven validity and reliability that evaluates various parameters of the manuscript [22]. The MHT which comprises six categories: legibility, shape, alignment, size, spacing, and typing speed has the validity and reliability in Turkish [22]. In the test application, children copy this string of words written on the paper onto the marked line just below. In the scoring stage, on the basis of 1/16 inch (0.15 cm) length, all deviations, the proportions between letters and the letters within themselves are evaluated by measuring this length with a ruler. The lowest score in each category of MHT is 0, and the highest score is 34 [22].

The Pediatric Quality of Life Inventory (PedsQL), developed by Varni et al., is widely used to assess participants’ health-related quality of life. Memik et al. (2007) conducted a study to establish the reliability and validity of the Turkish adaptation of the PedsQL. This 23-item instrument generates a total score ranging from 0 to 100, with higher scores indicating a better quality of life. PedsQL includes three main summary scores: Total Scale Score (TSS), Physical Health Summary Score (PHSS), and Psychosocial Health Summary Score (PsHSS), the latter encompassing emotional, social, and school functioning components (Belhan Çelik et al., 2022) [23]. The control group of the study consisted of children who did not receive any intervention. Children in the study group received cognitive activity training within the scope of cognitive rehabilitation method frequently used in the field of occupational therapy in addition to the secondary school they continued. As an intervention in the study group, the sessions were conducted two days a week for a total of 10 weeks (20 sessions). The initial assessments were administered to children in both groups. At the end of the 10 weeks, the evaluations were repeated in both groups.

#### Implementation of occupational therapy intervention

The study was conducted in two stages. In the first stage, a preliminary screening was carried out to identify refugee children aged 13–16 who had experienced war-related trauma, exhibited post-traumatic stress responses (PTSR), and showed signs of cognitive vulnerability. A pre-assessment comparing children with and without PTSR confirmed significantly lower cognitive performance in the PTSR group, justifying their inclusion in the intervention phase. Based on the full inclusion criteria, 34 eligible participants were selected.

The second stage involved the randomized controlled trial. Participants were evaluated using the Loewenstein Occupational Therapy Cognitive Assessment (LOTCA), Resiliency Scales for Children and Adolescents (RST), Mindful Attention Awareness Scale for Children (MHT), and the Pediatric Quality of Life Inventory (PedsQL). They were randomly assigned to two equal groups: the study group (*n* = 17), which received cognitive activity training based on occupational therapy principles, and the control group (*n* = 17), which continued with regular school activities without receiving any additional intervention.

##### Cognitive Activity Training (CAT)

The remedial intervention consisted of cognitive activity training delivered over 10 weeks (20 sessions, twice weekly). Each session lasted approximately one hour. The program was individualized based on the results of cognitive assessments (LOTCA), focusing on person-centered, occupation-based goals. The intervention was designed and implemented by the first author, a therapist with both clinical and academic expertise in cognitive rehabilitation, who also received doctoral-level training specifically in cognitive activity-based interventions for refugee populations.

Intervention content was structured around core cognitive domains such as attention, memory, planning, sequencing, organization, and visual-motor integration. Activities were graded in complexity and intensity to support progressive development of cognitive skills. Both individual and group-based sessions were used to apply targeted strategies in a holistic, activity-based format.

The intervention emphasized therapist-child collaboration to foster intrinsic motivation and engagement. Each participant followed a progressively structured plan moving from basic cognitive activities to tasks requiring higher-level executive functioning.

The cognitive retraining model, which is a widely used model in the field of cognitive rehabilitation was developed by Averbuch and Katz and is commonly used in adolescents and adults with neurological and psychological disorders [24]. As part of this model, LOTCA was developed and standardized and has become a valid and reliable cognitive skill assessment method used all over the world [20]. In addition to individual activities within the scope of cognitive training, children were accepted into the intervention environment as a couple in some sessions and a competitive environment was created between them. Thus, their motivation was kept high throughout the entire session [25]. The intervention consisted of play activities for various cognitive skills, and the games ranged from simple to complex in accordance with skill levels; It was planned to progress from basic skills such as attention-concentration to metacognitive strategies that include more complex executive skills. As a learning technique; cue/action and feedback techniques were used. In order to improve the children’s ability to create their own strategies during the games, an approach was not made to show the mistakes directly. Instead, “Why do you think it didn’t happen? What was missing? What should you do now to make up for the lack?” A method was used to activate thinking processes with questions such as [25].

The cognitive requirements of the activities in the games selected for children were examined; games that were suitable for the needs of the children were used. Practice-based trainings, which are frequently used in cognitive rehabilitation, focus on certain cognitive skills or involve the repetitive application of basic mental exercises on a computer, tablet, or phone [26]. Usually, these mental exercises are incorporated into various game scenarios and are usually placed in different game contexts. In our study, the intervention incorporated both board games played on a table and digital, technology-based games designed to enhance attention, memory, visual-spatial skills, and executive functions. The digital application automatically adjusted the difficulty level based on the child’s performance, modifying speed and task complexity speed and task complexity accordingly.

Along with the cognitive activities on printable cognitive therapy worksheets and game packages, an application technology was used named The MentalUp. This software, which is installed on the researcher’s computer, provides artificial intelligence-generated games and activities for the participant and determines each child’s level based on their responses to the tasks and continued under the supervision of a therapist in the last 20 min of the 1-hour session. MentalUp is an AI-powered cognitive training tool that offers age-appropriate exercises to enhance attention, memory, and problem-solving skills in children. This application is designed to enhance children’s cognitive and learning abilities by offering a variety of games that target key skills such as short-term memory, divided and sustained attention, reaction control, focus, mathematical reasoning, planning, organization, and visual processing. As players progress, the system dynamically adjusts the game difficulty using artificial intelligence to match their skill level and promote continuous improvement. Research has demonstrated the effectiveness of MentalUp in enhancing children’s cognitive functions [23].

###  Randomization approach

One of the authors, Gonca Bumin developed and securely kept a digital randomization list. Children who met the inclusion criteria were randomly assigned to the groups, with equal probability and independent of previous assignment, by means of computer software ([http://www.randomizer.org]).The participants were blinded to group assignment at baseline. The principal investigator, Sümeyye Belhan Çelik, managed participant enrollment, training design and implementation, data collection and analysis. The second investigator, Gonca Bumin, contributed to writing, reviewing, and editing. The study followed the CONSORT (Consolidated Standards of Reporting Trials) guidelines [27]. At baseline, Sümeyye Belhan Çelik, who made the evaluations, was blinded to the children’s group assignment.”

### Quantitative data analysis

All statistical analyses were conducted using IBM SPSS 21, and the significance level was set at *p* <.05. Before conducting group comparisons, assumptions related to data distribution and variance homogeneity were assessed. The Kolmogorov–Smirnov test was used for normality checks, while Levene’s test evaluated variance homogeneity. For categorical variables, the Pearson Chi-square tes**t** was applied to examine distribution differences across groups [28]. Additionally, the statistical analyses proposed for this project largely followed the principles and recommendations for controlled trials of randomized groups included in the CONSORT guide [27].

Interventions with pre- and post-design variables were evaluated using the One-Way Analysis of Covariance (ANCOVA) if assumptions were met, including normality, homogeneity of variances, and linear relationships. Clinical significance was determined for ANCOVA with partial eta-square (η2) according to Cohen’s suggested cut-off values (0.0099 small, 0.0588 medium, and 0.1379 large effect) [19]. The linear relationship between the dependent variable and the covariate was determined visually with a scatter plot, and the strength and direction of the relationship were determined with the Pearson correlation coefficient. Due to the violation of the regression slope homogeneity assumption in ANCOVA, a Mixed Design ANOVA was conducted to compare group means across selected variables. To explore the source of the difference, a Bonferroni-corrected Simple Effects analysis was conducted following the identification of a statistically significant interaction effect [28]. Descriptive statistics were also computed, with continuous variables summarized as means and standard deviations, while categorical data were reported using frequencies and percentages. The required sample size was determined using G*Power software (version 3.1.9.2), and a power analysis was conducted to ensure sufficient statistical power. Based on this analysis, a minimum of 17 participants per group was necessary to achieve 95% power with a significance level of α = 0.05, assuming an effect size of Cohen’s d = 0.50, which is commonly used to represent a medium effect size (Cohen, 1988).

## Results

### Preliminary screening to identify eligible participants

A sample of 82 Syrian refugee children (42 females, 40 males), aged between 13 and 16 years (M = 14.32, SD = 0.61), participated in the initial screening phase. All participants had been exposed to war in Syria. Post-traumatic stress reactions (PTSR) were assessed using the Child PTSD Reaction Index (CPTS-RI). The mean CPTS-RI score for children identified with PTSR (*n* = 34) was 30.58 (SD = 13.89), whereas for those without PTSR (*n* = 34), the mean was 6.82 (SD = 2.72).

To assess baseline cognitive performance, all 82 children were evaluated using the LOTCA. In Table 1, a comparison is presented between refugee children who were exposed to war in Syria and who either developed or did not develop post-traumatic stress reactions.

**Table 1:** Baseline cognitive differences between refugee children with and without post-traumatic stress

**Reaction prior to group assignment**	Groups (n=68)		
	Group without post-traumatic stress reaction		
	Group with post-traumatic stress reaction	Group without post-traumatic stress reaction	*P* values
LOTCA TOTAL	86.29±12.53	112.61±1.65	0.000*
LOTCA ORIENTATION	16.00±0.00	16.00±0.00	1.000
LOTCA VISUAL PERCEPTION	11.52±2.67	15.44±0.99	0.000 *
LOTCA SPACE PERCEPTION	9.20±2.01	11.50±0.78	0.000 *
LOTCA PRAXIS	10.64±1.09	11.00±11.64	0.000 *
LOTCA VISUAL-MOTOR ORGANIZATION	19.52±3.90	19.00±27.35	0.000 *
LOTCA THINKING PROCESS	21.64±4.44	21.50±30.67	0.000 *
LOTCA ATTENTION	3.50±0.50	3.50±3.76	0.025 *

*LOTCA* Loewenstein Occupational Therapy Cognitive Assessment

*Statistical significance was found (p<0.05)

A non-parametric Mann–Whitney U test was conducted to compare cognitive performance between the PTSR and non-PTSR groups. As shown in Table 1, children with PTSR scored significantly lower in all LOTCA subdomains except for orientation. These results supported the rationale for including only those children who had both trauma exposure and measurable cognitive vulnerability in the main intervention study. Consequently, 34 children with PTSR who also met the other inclusion criteria were randomly assigned to intervention (*n* = 17) and control (*n* = 17) groups for the experimental phase.

### Main findings

#### Sociodemographic findings

Thirty-four refugee children, 17 in the study group and 17 in the control group, participated in our study. Of the 34 children, 17 (50%) were male and 17 (50%) were female. Table 2 shows that the gender distribution in both groups was homogeneous (*p* = 0.732). The mean age of the participants was 14.35 ± 0.73 years. The average number of siblings is 2.94 ± 1.96 and the average of the year they resided in Türkiye is 7.47 ± 2.23. There was no statistically significant difference between the mean age (*p* =.525), number of siblings (*p* = 1.000), years of residence in Türkiye (*p* =.944), PTSD (*p* =.341) in the two groups. F) Comparison of demographic data and diagnostic variable distributions of the groups is shown in Table 2. This table shows us that the two groups are homogeneous.

**Table 2:** Comparison of demographic information and distributions of the groups

		Study Group (*n* = 10)	Control Group (*n* = 10)	Test Statistics
Sex	Female	8(47.10%)	9(52.90%)	χ^2^ = 0.118* *p* =.732
	Male	5(52.90%)	8(47.10%)	
Age (year) (Mean ± SD)		14.29 ± 0.84	14.41 ± 0.61	u = 128.50*** *p* =.525
Residence (year) (Mean ± SD)		7.58 ± 2.06	7,35 ± 2,44	u = 142.50*** *p* =.944
CPTS-RI		32.76 ± 15.98	28,11 ± 11,71	t = 0.967* *p* =.341

*SD* Standard deviation, *CPTS-RI* Child Post Traumatic Stress Disorder Reaction Index Scale, *MoCA*Montreal Cognitive Assessment

*: Pearson chi-square test statisitc value

**: Student’s t test statisitc value

***: Mann-Whitney U test statisitc value

#### Cognitive skills findings

Cognitive skills of individuals were evaluated with LOTCA. Cognitive skill values were obtained in 6 sub-areas: visual perception (VP), spatial perception (SP), motor praxis (MP), visual-motor organization (VMO), thinking processes (TP) and attention. To assess the effectiveness of the intervention while controlling for initial differences between groups, ANCOVA was conducted using pre-test scores as covariates (Table 3). This allowed for a more accurate comparison of post-test outcomes by adjusting for baseline variability.

**Table 3:** Findings from ANCOVA Analysis

**Measure**		**M (SD)**		**Test Statistics Values**		
		**A**	**B**	**Groupa**	**Covariateb**	**Interaction Effectc**
LOTCA Cognitive Assessment						
LOTCA Total	1	87.00 (11.23)	85.58 (14.03)	F(1.31)=28.211 p<.001* ɳ2=.476	F(1.31)=64.923 p<.001*	F(1.30)=4.003 p=.054
	2 3	102.76 (5.70) 102.48	86.70 (7.73) 86.98			
Visual Perception	1	11.94 (2.41)	11.11 (2.93)	F(1.31)=96.670 p<.001* ɳ2=.757	F(1.31)=39.274 p<.001*	F(1.30)=0.166 p=.687
	2 3	14.05 (1.78) 13.85	11.05 (2.68) 11.40			
Academic Performance						
MHT Total	1	174.41 (11.91)	175.29 (12.75)	F(1.31)=23.400 p<.001* ɳ2=0.430	F(1.31)=620.515 p<.001*	F(1.31)=3.920 p=.057
	2 3	178.58 (13.16) 179.06	174.82 (12.06) 174.41			
PedsQL						
EFS	1	57.35 (12.00)	60.58 (11.97)	F(1.31)=12.586 p=.001* ɳ2=0.289	F(1.31)=68.584 p<.001*	F(1.30)=2.026 p=0.165
	2 3	64.70 (11.10) 65.75	59.41 (11.30) 57.94			
SFS	1	58.82 (8.39)	55.58 (8.27)	F(1.31)=44.343 p<.001* ɳ2=.589	F(1.31)=56.557 p<.001*	F(1.30)=0.946 p=.339
	2 3	67.35 (54.70) 66.35	57.70 (7.80) 56.00			
SsFS	1	57.35 (13.00)	53.23 (14.46)	F(1.31)=20.342 p<.001 ɳ2=.396	F(1.31)=29.151 p<.001*	F(1.30)=3.307 p=.079
	2 3	68.52 (12.71) 67.51	47.94 (17.41) 49.99			

*N *34, *M *Mean, *SD *Standard deviation, *1* Before training, *2 *After training, *3 *Adjusted mean of post-test scores, *A S*tudy group, *B *Control group, *ɳ*2 Affect size,* LOTCA* Loewenstein Occupational Therapy Cognitive Assessment, *VP *Visual Perception, *MHT *Minnesota HandWriting Test, *PedsQL *Pediatric Quality of Life Inventory, *EFS *Emotional Functioning Score, *SFS *Social Functioning Score, *SsFC* School Functioning Score

*Statistical significance was found (p<.05); ANCOVA=analysis of covariance

aColumn comparing the adjusted mean of posttest in groups; bColumn where the standard regression coefficient is evaluated; cColumn where homogeneity in regression coefficients is evaluated

Before running ANCOVA, we checked two essential assumptions: linearity between covariates and dependent variables and homogeneity of regression slopes. It was observed that the linear relationship (*r* >.30) between all dependent variables and covariates for LOTCA Total and LOTCA VP were higher than 0.30 (respectively; *r* = 0,51; *r* = 0,76). The assumption of homogeneity of regression coefficients for all variables in Table was evaluated with the interaction effect, and it was found that they are homogeneous (respectively; F (1.30) = 4.003, F (1.30) = 0.166; *p* >.05).

ANCOVA findings for MWT revealed that the pre-test score averages for two variables in Table 3 were held constant (respectively; 86.29, 11.53), and the post-test adjusted means of LOTCA Total and LOTCA VP in the study (respectively; 102.48, 13.85) and control (86.98, 11.40) groups were compared. LOTCA total and LOTCA VP were found to be statistically significant differences in the post-intervention study group adjusted mean compared to the control group (respectively; (F(1.31) = 28.211, *p* <.00; F(1.31) = 96.670, *p* <.001). Based on these results, the children in the study group had improved LOTCA VP and LOTCA total scores.

Mixed-design ANOVA results revealed statistically significant interaction effects in the LOTCA subdimensions, specifically in SP, MP, VMO, TP, and Attention scores (respectively; F(1.32) = 32.099, *p* <.001; F(1.32) = 13.74, *p* =.001, F(1.32) = 63.566, *p* <.001; F(1.32) = 58.863, *p* <.001; F(1.32) = 24.123, *p* <.001) (Table 4). The mean scores over time in these areas for individuals in the study and control groups showed a significant difference. An analysis of the source of this difference revealed no statistically significant difference between the pre-intervention mean scores of the two groups in these variables. For the within-group part of the interaction effect, post-intervention scores significantly improved in the study group (*p* <.001), with no significant changes observed in the control group (Table 4).

**Table 4:** Findings from Mixed-Design ANOVA Analysis

Measure	M (SD)				Interaction Effect	Pairwise Comparisons	
	1		2			Within Group	Between Groups
	A	B	A	B			
LOTCA Cognitive Performance							
SP	9.23 (1.75)	11.05 (1.02)	9.17 (2.29)	8.88 (2.34)	F(1.32)=32.099, p<.001*, ɳ2=.501	A: 1<2, p<.001*	(1): A>B, p>.05
MP	10.70 (1.04)	11.53 (0.51)	10.58 (1.71)	10.53 (0.94)	F(1.32)=13.74, p=.001*, ɳ2=.300	A: 1<2, p<.001*	(1): A>B, p>.05
VMO	19.47 (3.90)	24.17 (2.83)	19.58 (4.03)	19.41 (3.80)	F(1.32)=63.566, p<.001*, ɳ2=.665	A: 1<2, p<.001*	(1): A<B, p>.05
TP	20.76 (4.13)	26.11 (2.87)	22.53 (4.69)	21.76 (4.11)	F(1.32)=58.863, p<.001*, ɳ2=.648	A: 1<2, p<.001*	(1): A<B, p>.05*
Attention	3.41 (0.50)	4.00 (0.00)	3.58 (0.50)	3.41 (0.50)	F(1.32)=24.123, p<.001*, ɳ2=.430	A: 1<2, p<.001*	(1): A<B, p>.05
Academic Performance							
RST Total	97.22 (27.49)	107.66 (27.27)	101.11 (27.46)	99.26 (25.22)	F(1.32)=12.264, p<.001*,ɳ2=.277	A: 1<2, p<.001*	(1): A<B, p>.05
PedsQL							
PHSS	66.45 (11.16)	78.45 (9.69)	64.74 (11.93)	62.74(12.62)	F(1.32)=20.646, p<.001*, ɳ2=.392	A: 1<2, p<.001*	(1): A<B, p>.05
PsHSS	58.13 (8.61)	65.88 (7.47)	56.46 (7.65)	54.01 (8.89)	F(1.32)=21.292, p<.001*, ɳ2=.400	A: 1>2, p<.001*	(1): A<B, p>.05
Total	62.29 (9.22)	73.09 (7.04)	60.60 (8.65)	58.38 (9.85)	F(1.32)=42.022, p<.001*, ɳ2=.568	A: 1>2, p<.001*	(1): A<B, p>.05

A simple effects analysis with Bonferroni correction was used. bF test value

*N* 34, *M *Mean, *SD *Standard deviation; study group, n=17; control group, n=17. A=study group; B=control group; 1=before training; 2=after training. ɳ2= affect size, *LOTCA* Loewenstein Occupational Therapy Cognitive Assessment; *SP *Spatial Perception, *MP *Motor Praxis, *VMO *Visual-Motor Organization, *TP *Thinking Process *RST* Reading Speed Test, *PedsQL* Pediatric Quality of Life Inventory, *PHSS *Physical Health Summary Score: *PsHSS *Psychosocial Score, *ANOVA *Analysis of variance

*Statistical significance was found (p<.05)

#### Academic performance findings

Reading Speed Test (RST) and Minnesota Writing Test (MWT) were used to assess the individuals’ academic performance in two distinct categories: reading speed and writing abilities. Mixed Design ANOVA findings revealed that, the RST results indicated statistically significant interaction effects (F(1.32) = 12.264, *p* <.001) (Table 4). An analysis of the source of this difference revealed no statistically significant difference between the pre-intervention mean scores of the two groups in these variables. For the within-group part of the interaction effect, post-intervention scores significantly improved in the study group (*p* <.001), with no significant changes observed in the control group (Table 4).

The mean scores over time in this test for individuals in the study and control groups showed a significant difference. An analysis of the source of this difference revealed no statistically significant difference between the pre-intervention mean scores of the two groups in these variables. For the within-group part of the interaction effect, post-intervention scores significantly improved in the study group (p<.001), with no significant changes observed in the control group (Table 4).

When the MWT scores were examined, it was observed that the linear relationship (*r* >.30) between all dependent variables and covariates for MWT was higher than 0.30 (respectively; *r* =.51; *r* =.76). The assumption of homogeneity of regression coefficients for all variables in Table 3 was evaluated with the interaction effect, and it was found that they are homogeneous (respectively; F(1.31) = 3.920, F (1.30) [, ]; *p* >.05).

Statistical analysis using ANCOVA for MWT showed that, the pre-test score averages for all variables in Table 3 were held constant (174.85), and the post-test adjusted means of MWT in the study (179.06) and control (174.41) groups were compared. MWT scores showed statistically significant differences in the post-intervention study group adjusted mean compared to the control group (F(1.31) = 23.400, *p* <.001). Based on these results, the children in the study group had improved writing skills.

#### Quality of life findings

Examining the PsHSS in PedsQL’s sub-dimensions emotional functioning score (EFS), social functioning score (SFS) and school functioning score (SsFS), a test that assesses quality of life and meets ANCOVA assumptions, it was observed that the linear relationship (*r* >.30) between all dependent variables and covariates was higher than 0.30 (*r* = 0.76).

The assumption of homogeneity of regression coefficients for all variables in Table 3 was evaluated with the interaction effect, and it was found that they are homogeneous (respectively; F(1.30) = 2.026, F(1.30) = 0.946, F(1.30) = 3.307, *p* >.05). Mixed Design ANOVA findings revealed that, the pre-test score averages for three variables in Table 3 were held constant (respectively; 58.97, 57.20 and 55.29) and the post-test adjusted means of EFS, SFS and SsFS in the study (respectively; 65.75, 66.35, and 67.51) and control groups (57.94, 56.00 and 49.99) were compared.

Significant differences were found in the adjusted post-intervention mean scores of the study group compared to the control group (F (1.31) [, ] = 12.586, *p* <.001; F (1.31) [, ] = 44.343, *p* <.001; F (1.31) [, ] = 20.342, *p* <.001). Based on these results, improvements were observed in the study group’s emotional, social, and school functioning related to quality of life (Table 3).

Mixed-design ANOVA findings indicated that, interaction effects were statistically significant in the PedsQL Total and its sub-dimensions Physical Health Summary Score (PHSS) and Psychosocial Health (PsHSS = EFS + SFS + SsFS) scores (respectively; F(1.32) = 42.022, *p* <.001; F(1.32) = 42.022, *p* =.001; F(1.32) = 20.646, *p* <.001; F(1.32) = 21.292, *p* <.001) (Table 4). The mean scores over time in these areas for individuals in the study and control groups showed a significant difference. An analysis of the source of this difference revealed no statistically significant difference between the pre-intervention mean scores of the two groups in these variables.

For the within-group part of the interaction effect, the mean scores of the study group after the training were significantly different from pre-intervention (*p* <.001). In contrast, the control group showed no statistically significant changes (Table 4). Based on these results, the children in the study group’s PedsQL Total score, physical health total score and psychosocial total score improved (Table 3).

**Fig. 2:**
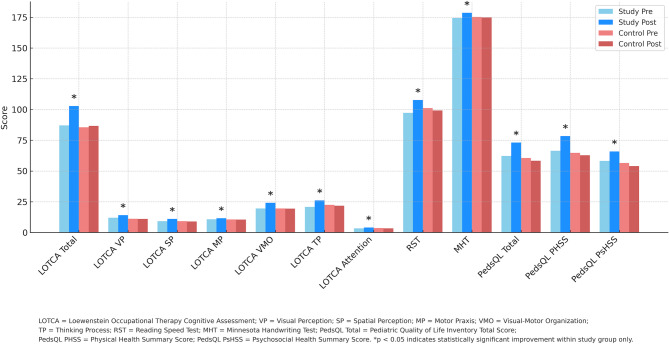
Pre- and post-test comparison of cognitive, academic, and quality of life outcomes in study and control groups

A visual summary of all outcome measures for the study and control groups, both pre- and post-intervention, is provided in Figure 2. The figure presents changes in cognitive performance, academic performance, and quality of life measures. Statistically significant improvements (*p* <.05) were observed only in the study group, as indicated by asterisks (Figure 2).

## Discussion

The primary objective of this study was to examine the effects of school-based cognitive activity training on cognitive skills, academic performance and quality of life in refugee children. Three main findings have been put forward: first, the study suggested that cognitive training intervention in the school may be effective in enhancing cognitive skills in refugee children who have a high level of post-traumatic stress reaction. At the end of the 10-week, 20-session program, the study group showed significant improvements in multiple areas. These included cognitive functions (such as visual perception, spatial perception, praxis, visuomotor-organization, thinking operations, and attention), academic performance (reading speed and writing), and quality of life domains (including psychosocial; emotional; social; school functioning; and physical health).

These results are in line with previous research, which has shown that refugee and immigrant children exposed to trauma often perform lower on standardized cognitive assessments [10, 11, 29]. While cognitive therapy for trauma typically addresses emotional symptoms, few interventions directly target cognitive skill-building in refugee populations. Thus, it is believed that CAT can enhance cognitive skill development as well as academic abilities and quality of life, all of which are critical for children’s academic achievement.

In the literature, most cognitive interventions have been directed at neurodevelopmental disorders rather than trauma-exposed refugee populations [26, 29, 30]. Although cognitive-behavioral therapy (CBT) has shown positive outcomes in refugee children by reducing PTSD and depression symptoms [31, 32], no research has demonstrated the efficacy of cognitive skills training for this population. For instance, a 10-session school-based CBT group intervention for sixth-grade immigrant children and a 23-session trauma-focused program implemented by Layne et al. for war-affected Bosnian adolescents both demonstrated significant reductions in PTSD and depression symptoms, highlighting the effectiveness of structured, psychoeducational, and cognitive-behavioral group therapies in trauma-exposed youth populations [33, 34]. As can be observed from these studies, the research conducted with refugee children focused on CBT, and no studies were discovered on the effects of cognitive skills training. This study is the first randomized controlled evidence-based study conducted on refugee children. It is thought that the reason why so few studies have been conducted on the refugee population is because the pre-war, wartime and post-war resettlement experiences of refugee children are heterogeneous and the erosion caused by refugee status is multifactorial.

Play-based activities, such as board games, have been shown to support cognitive development in children [35, 36]. Building on this foundation, our findings support the idea that multi-content cognitive interventions that incorporate metacognitive strategies, structured tasks, and interactive formats—such as board and digital games—may yield significant improvements in both cognitive and psychosocial domains, particularly in trauma-exposed populations where both cognitive and emotional needs must be addressed [37, 38].

In line with this, our study deliberately utilized a hybrid model that combined tabletop and technology-based cognitive tasks. By incorporating varied formats and skill targets, the intervention aimed to stimulate motivation, adaptive thinking, and broader developmental gains. This multi-content structure may explain the observed improvements across several cognitive domains and quality of life indicators in the intervention group.

A prior study involving refugee children indicated frequent maltreatment during migration and low motor and process skill levels [39]. In our study, children with post-traumatic stress symptoms exhibited significantly lower praxis scores. It is likely that game-based sequencing tasks in our intervention contributed to improvements in praxis by supporting activity planning and execution. Praxis involves multiple cognitive and motor abilities—such as attention, error detection, and self-correction—and is crucial for functional performance [40].

Although no previous studies have specifically investigated praxis skills in refugee populations, research indicates that these children often display lower motor development due to environmental and traumatic factors compared to typically developing peers [40, 41]. Given the strong association between motor and cognitive development [42], evaluating both domains in refugee children within a unified framework may provide deeper insight into their developmental needs.

In our study, the academic performance of refugee children in both the study and control groups was assessed using basic reading and writing tasks. At the conclusion of the 10-week intervention, a significant improvement was observed in the academic performance of children who received cognitive skills training, particularly in reading and writing skills.

Academic success is influenced by a variety of factors, including cognitive abilities, motivation, anxiety, and family dynamics such as parenting strategies [43–46]. Among refugee children, trauma linked to forced migration has been shown to adversely impact academic performance by impairing attention, executive functions, memory, and social engagement [10, 47]. Our findings indicate that CAT, by enhancing core cognitive abilities, can indirectly support academic skill acquisition.

Prior research has emphasized the value of structured cognitive interventions in supporting educational outcomes for refugee populations [23]. However, programs specifically targeting the academic dimension—especially through cognitive skill-building—remain limited. Our study addresses this gap, providing randomized controlled evidence of CAT’s role in improving literacy-related outcomes.

Reading speed is a critical academic skill that depends on the rapid scanning and processing of visual information. Efficient readers activate complex sensory and cognitive mechanisms to recognize letter patterns and derive meaning from text. This process integrates visual attention, working memory, and long-term memory functions [48]. Research indicates that strengthening visual-spatial perception and attentional capacity can significantly improve reading performance [49]. Similarly, classification abilities—such as organizing objects by shape or color—are positively associated with literacy skills [50]. In our study, cognitive gaming activities targeting visual attention, visual scanning, spatial perception, and categorization contributed to notable improvements in visual perception, spatial perception, visual-motor organization, and thinking processes. These gains likely played a role in enhancing the children’s reading speed.

Moreover, although reading fluency alone does not encompass the full scope of academic reading competence, it has been widely accepted as a core component of early academic functioning and is a strong predictor of comprehension in both primary and secondary education settings [51]. Given the contextual limitations of working with refugee adolescents in a school-based intervention, reading speed was used as a feasible and ecologically valid proxy for basic academic skill performance. These findings should be interpreted as reflecting improvements in basic academic skills (e.g., reading speed and handwriting), rather than overall academic performance.

Handwriting, is another essential academic function. It involves the coordination of perceptual-motor, cognitive, and tactile-kinesthetic abilities [52]. Effective handwriting requires fine motor control, bilateral integration, praxis, and sustained attention. Problems in these areas often result in slow, disorganized, or illegible writing [53]. In our study, the MHT assessment showed that children in the intervention group improved significantly in handwriting performance after the training.

Visual-motor abilities—crucial for tasks like handwriting—are considered strong predictors of academic success, especially during early school years [14, 54]. Improvements in visual-motor organization (VMO), as observed in our intervention group, suggest that CAT may contribute to academic development through enhanced motor coordination and spatial awareness [55]. This finding aligns with previous school-based occupational therapy programs that improved handwriting legibility and visual-motor skills by focusing on cognitive and manual dexterity training [56].

These components reflect the mechanical aspects of writing—such as legibility, form, alignment, size, spacing, and speed—rather than the broader dimensions of written expression, including grammar, organization, and content development. However, these mechanical elements are foundational to written academic performance, especially in school settings where fluency and legibility directly affect students’ ability to complete classroom tasks efficiently. Research has also operationalized writing competence through such observable features of handwriting when assessing academic skills in children [57]. Therefore, while our assessment did not encompass the full range of written expression, it still provides meaningful insight into one important dimension of academic performance.

Alongside interventions, it’s crucial to note that despite language acquisition efforts, refugee children may still experience difficulties in reading and writing due to structural differences between languages and incomplete adaptation to Turkish. These challenges underline the need for integrated cognitive and language-focused strategies [58].

In this context, enhancing refugee children’s reading and writing skills requires not only cognitive education but also appropriate instructional strategies and, when necessary, preparatory language support. Supplementary audiovisual materials have also been suggested to improve learning outcomes among Syrian students adapting to Turkish [59].

School-based screening of cognitive and perceptual abilities is essential to identify developmental concerns early and initiate timely intervention programs, especially during preschool years [14]. Such initiatives can strengthen academic foundations and address trauma-related challenges.

School-based rehabilitation is noted to improve mental health issues associated with trauma, diminish emotional and behavioral symptoms, and enhance school adaptation [60]. While the intervention was implemented in a school setting and facilitated engagement, further research is needed to examine feasibility, scalability, and long-term sustainability of school-based implementation in diverse settings.

Although CBT has been widely applied to reduce trauma symptoms in refugee populations and has shown improvements in mental health and quality of life [61, 62], no studies have specifically explored the impact of cognitive skills training on these outcomes. Our study fills this gap by demonstrating that a school-based cognitive activity intervention significantly enhanced PedsQL scores among refugee adolescents.

The improvements observed across sub-dimensions—including social functioning, school functioning, emotional well-being, and physical health—may be attributed to the intervention’s engaging, play-based structure and the supportive social interactions it promoted. The use of collaborative pair games and rule-based activities provided children with opportunities to communicate, share, and build peer relationships, especially valuable for those lacking social support [63]. This likely contributed to the substantial effect size seen in social functioning scores. The integration of structured game-based cognitive tasks in this study may have facilitated engagement and enjoyment, which could have supported improvements. However, generalizations regarding broader therapeutic benefits of play require further empirical evidence.

Our study showed significant results on cognitive activity training in the emotional and physical health sub-dimensions. Such outcomes may support children’s ability to regulate emotions and participate more confidently in daily routines within school and social environments.Consequently, participation is seen as a fundamental objective of pediatric rehabilitation and positively impacts quality of life [64]. In our study, over a period of 10 weeks, children engaged in pleasurable social interactions with both the therapist and their peers in the school setting, meeting twice weekly.

The results suggest that enhancing cognitive skills may play an important role in supporting the development of refugee children. Given that our intervention incorporated play and free time, critical occupational domains often absent in children who have experienced trauma, the study group demonstrated improvements across all sub-dimensions of quality of life, as reflected in the PedsQL scores. Furthermore, in our school-based study, the supportive influence of the school environment, where children are familiar and interact with their peers in a secure setting, may have contributed to the positive outcomes observed in quality of life indicators.

Alongside cognitive skills training, we suggest the design of individual and group psychosocial intervention studies within the school setting to enhance refugee children’s coping mechanisms for post-traumatic stress, social skills, and overall quality of life, which may provide promising results.

There are some limitations in our study. It is important to note that refugees included in our study only living in Istanbul, Türkiye’s largest city, among the participants residing in Türkiye. While our study encompassed individuals from diverse demographic backgrounds, the presence of refugee children living in Istanbul could restrict the applicability of our findings. Moreover, the study was conducted at a single center and may not reflect experiences in other institutional or regional settings. Future studies involving refugee children from other geographic regions, including rural or underserved areas, would help increase the generalizability and contextual relevance of the findings.

One of the limitations of this study is the potential influence of non-specific factors such as increased attention and therapist interaction experienced by the intervention group. Although participants were randomly assigned and blinded to group allocation at baseline, blinding could not be maintained during the post-intervention assessments. Participants were aware of whether they received the intervention, which may have influenced their motivation or performance during the follow-up measurements. While the control group received no additional support outside their regular school curriculum, the intervention group may have benefited from increased engagement and individualized attention. Future studies may consider the use of attention-matched active control groups or placebo-like interventions to more clearly isolate the specific effects of cognitive activity training. In future trials, the inclusion of attention-matched active control groups—such as structured non-cognitive or recreational sessions—may help to more clearly isolate the specific effects of cognitive activity training from general attention effects or novelty-related engagement.

In addition, the lack of access to age-specific normative data for the standardized assessment tools used (e.g., LOTCA, MWT) presents another important constraint. Because these instruments were not originally standardized for adolescents aged 13–16—especially not for war-exposed refugee populations—the interpretation of results in relation to typical developmental expectations is limited. Furthermore, due to the lack of standardized instruments developed specifically for culturally diverse or trauma-affected refugee populations, the cultural validity and contextual appropriateness of the measures may be limited. Although we relied on relative changes and group comparisons to assess improvement, future studies would benefit from the inclusion of localized or age-adjusted normative benchmarks to strengthen interpretability and clinical inference.

Another limitation concerns the operationalization of academic performance in the current study. The term “academic performance” was initially used in a broad sense; however, the assessments conducted focused only on two basic components—reading fluency and handwriting legibility. Higher-order academic skills such as reading comprehension, written expression, and subject-specific knowledge were not directly evaluated. Future studies should consider incorporating broader academic outcome measures aligned with age-specific educational expectations.

Lastly, the small sample size reduces statistical power and limits the robustness of subgroup analyses. Future studies with larger and more heterogeneous samples would provide greater insight into variability across individuals. Additionally, the absence of long-term follow-up data limits our ability to assess the sustainability of the observed improvements. Future longitudinal research is needed to determine whether gains in cognitive skills, academic performance, and quality of life are maintained over time. Future studies are also recommended to include structured follow-up assessments to evaluate the persistence of cognitive, academic, and quality of life improvements over time.

## Conclusion

This study provides the first randomized controlled evidence that school-based cognitive activity training (CAT) can significantly improve cognitive functions, academic performance and multiple domains of quality of life among refugee adolescents. The intervention incorporated play-based, engaging, and developmentally appropriate tasks that align with occupational therapy’s emphasis on participation and functional skill-building.

Given that cognitive challenges and trauma-related difficulties often co-occur in displaced populations, early school-based interventions such as CAT may offer a promising avenue for addressing both academic and psychosocial needs. The structured nature of the program, along with peer interaction and familiar school settings, likely facilitated the observed improvements in emotional, social, and school functioning as measured by PedsQL scores.

Alongside cognitive skills training, we suggest the design of individual and group psychosocial intervention studies within the school setting to enhance refugee children’s coping mechanisms for post-traumatic stress, social skills, and overall quality of life, which may provide promising results. These interventions should be trauma-informed, culturally sensitive, and implemented in a way that ensures continuity and sustainability.

Furthermore, school-based screening of cognitive and perceptual abilities is essential to identify developmental concerns early and initiate timely intervention programs, especially during preschool years. Such initiatives not only address trauma-related challenges but also support stronger academic foundations, contributing to long-term educational and psychosocial adjustment.

Future studies should explore the scalability of this intervention, test long-term sustainability, and investigate its applicability across different cultural and educational contexts. Expanding the scope to include additional academic and behavioral outcomes, as well as incorporating qualitative perspectives, will further enrich the understanding of CAT’s impact on vulnerable child populations.

## Supplementary Information

Supplementary Material 1.

## Data Availability

Any data and materials associated with this article will be made available upon reasonable request to the corresponding author.

## Abbreviations

ANCOVA: Analysis of Covariance
ANOVA: Analysis of Variance
CAT: Cognitive Activity Training
CPTS-RI: Child Post Traumatic Stress Disorder Reaction Index
EFS: Emotional Functioning Score
LOTCA: Loewenstein Occupational Therapy Cognitive Assessment
MHT: Minnesota Handwriting Test
MoCA: Montreal Cognitive Assessment
MP: Motor Praxis
PedsQL: Pediatric Quality of Life Inventory
PHSS: Physical Health Summary Score
PsHSS: Psychosocial Health Summary Score
PTSD: Post-Traumatic Stress Disorder
RST: Reading Speed Test
SFS: Social Functioning Score
SP: Spatial Perception
SsFS: School Functioning Score
TP: Thinking Process
VMO: Visual-Motor Organization
